# Spiritual Well-Being and Hopelessness Levels in Individuals Who Have Experienced Myocardial Infarction in Turkey

**DOI:** 10.1007/s10943-024-02234-x

**Published:** 2025-01-07

**Authors:** Efe Seher, Bahar Çiftçi

**Affiliations:** Department of Fundamental of Nursing, Ataturk University, 25000 Erzurum, Turkey

**Keywords:** Nursing, Myocardial infarction, Spiritual well-being, Hopelessness

## Abstract

This study aimed to investigate the relationship between spiritual well-being and hopelessness levels in individuals who have experienced myocardial infarction. The study, which was descriptive and exploratory, was conducted on patients who had experienced myocardial infarction and undergone angiography in the coronary intensive care unit and cardiology ward of Ağrı Education and Research Hospital. The population of the study consisted of 151 patients who had experienced myocardial infarction and undergone angiography between December 2022 and February 2023. Data were collected using a Demographic Information Form, Spiritual Well-Being Scale, and Beck Hopelessness Scale. This study highlights the significance of the transcendent subscale of the Spiritual Well-Being Scale, as it is unaffected by indicators of mental health. Findings revealed that higher transcendent subscale scores were associated with lower hopelessness levels in individuals who had experienced myocardial infarction. Body mass index, family type, marital status, and fulfilling religious duties when healthy were significantly associated with spiritual well-being and hopelessness. These results underscore the importance of considering transcendent aspects when evaluating spiritual well-being in this population. Furthermore, a strong negative correlation was found between spiritual well-being and hopelessness levels in individuals who had experienced myocardial infarction. Higher levels of spiritual well-being were significantly associated with lower levels of hopelessness. This study provides valuable insights into the relationship between spiritual well-being and hopelessness in post-myocardial infarction patients. However, limitations relevant to this study, including its single-center design and cross-sectional nature, are also noted.

## Introduction

Myocardial infarction (MI) occurs when the coronary arteries become blocked or narrowed due to thrombosis or atherosclerosis, resulting in insufficient blood supply to the myocardium (Güven & Kantarcı, 2018). MI is a significant public health problem due to its potential to cause death and chronic diseases (Olgun et al., 2016). According to TUIK data, MI is the most important cause of death among cardiovascular diseases, with a rate of 37.7% (TÜİK, 2018). The disease seriously affects the lives of patients and their families after diagnosis and significantly disrupts their living conditions (Dural & Sarıtaş, 2017). With the increase in life expectancy worldwide, the burden on the healthcare system and care costs has also significantly increased. Effective disease management and continuity through teamwork can reduce these costs. The aim of managing these diseases is to increase the patient’s self-management of the disease, increase their independence, prevent mental illness, improve their quality of life, and prevent repeated hospitalizations (İncirkuş & Nahcivan, 2015).

Spiritual well-being is an important indicator of quality of life, and the transcendent subscale stands out as a unique component that is unaffected by mental health indicators (Ekşi et al., 2019). It enhances coping and interaction in stressful processes and improves psychosocial aspects (Soleimani et al., 2017). Studies have shown that post-MI lifestyle changes positively affect life span and quality of life. At the same time, problems related to recovery, sleep, disrupted social relationships, sexual changes, and anxiety negatively affect quality of life. Another condition affected by MI is hopelessness (Olgun et al., 2016).

There is a strong relationship between mental health and overall health, and spirituality is believed to affect mental health positively. Patients who experience cognitive problems such as stress, depression, and hopelessness may inevitably face tachycardia, ventricular fibrillation (VF), or recurrent MI after post-MI. Spirituality, a strong source of hope, is an inevitable component of holistic nursing care. Nurses should investigate the sources of hopelessness in post-MI individuals and provide professional support by valuing their beliefs and values (Yaman & Muz, 2021). This research makes a significant contribution by emphasizing the relationship between mental well-being and hopelessness levels after myocardial infarction (MI), which profoundly affects daily life activities. MI is a serious health problem that can be life-threatening and lead to chronic diseases. It can seriously affect the lives of patients and their families and significantly disrupt their living conditions.

This research is essential to understand the difficulties that post-MI patients face in their daily living activities and the relationship between these difficulties and their psychological well-being and hopelessness levels. While post-MI patients often face lifestyle changes, they may struggle with problems such as sleep, social relationships, sexual changes, and anxiety during the recovery process. These difficulties can affect patients’ mental well-being and hopelessness levels. Additionally, this research is essential to understand how the relationship between mental well-being and hopelessness levels may affect the daily quality of life of post-MI patients. While mental well-being can make it easier to cope with stressful processes, hopelessness levels can negatively affect the quality of life. Therefore, understanding this relationship may help identify interventions needed to support the holistic well-being of post-MI patients.

Therefore, this study aimed to investigate the relationship between spiritual well-being and hopelessness levels in individuals who have experienced myocardial infarction.

## Research Questions

Q1. What is the level of spiritual well-being in individuals who have had a myocardial infarction?

Q2. What is the level of hopelessness in individuals who have had a myocardial infarction?

Q3. Is there a relationship between the level of spiritual well-being and the level of hopelessness in individuals who have had a myocardial infarction?

## Materials and Methods

### Type of Research

This study is descriptive and exploratory, focusing on examining the association between spiritual well-being and hopelessness in individuals who have experienced myocardial infarction.

### Place and Time of Research

The research was conducted between December 2022 and February 2023 in the coronary intensive care unit (ICU) and cardiology unit of Ağrı Education and Research Hospital. Three specialist cardiologists work in the hospital. There are 23 beds in the coronary ICU and cardiology unit.

### Population and Sample of the Research

The population of the research consisted of patients who were diagnosed with myocardial infarction, underwent coronary angiography, and were admitted to the coronary ICU and cardiology unit. The research sample consisted of 171 patients who agreed to participate, met the research criteria, and were 18 or older.

Sufficient sample size was calculated using the G POWER 3.1.9.7 package program to determine the sample size. Since no similar study was found in the literature, a priori power analysis was performed, assuming a medium effect size of 0.3. Based on an alpha of 0.05 and a power of 0.95, the power analysis determined that the sample size should be 171 (Cohen, 2013). However, due to 20 patients providing incomplete responses, the research was completed with 151 patients.

### Inclusion Criteria for the Study

- Having had a myocardial infarction.
- Having undergone coronary angiography and stabilized.
- No communication problems.
- Being able to understand and speak Turkish.
- Being 18 years of age or older.

### Data Collection Tools

Data were collected using a Demographic Information Form, the Spiritual Well-Being Scale, and the Beck Hopelessness Scale. In the study, the Spiritual Well-Being Scale was used. The transcendent subscale of this scale was considered a key component in understanding the relationship between spiritual well-being and hopelessness levels (Bambling, 2024, Koenig & Carey, 2024).

### Demographic Information Form

The form consists of 6 questions on gender, age, BMI, marital status, family type, and performing religious duties when healthy to obtain information on patients’ descriptive characteristics, prepared by the researchers in line with the literature.

### Spiritual Well-Being Scale

Ekşi and Kardaş developed the Spiritual Well-Being Scale for adults, which consists of 29 items and three sub-dimensions (Ekşi & Kardaş, 2017). The scale consists of 29 items and includes “The feeling of belonging to a strong divine entity comforts me. I feel God’s help when I face a problem. My faith shows me a better way of life. My faith and values strengthen my ability to withstand hardships. I think events beyond my control cause my distress. etc.” The sub-dimensions are Transcendence, Harmony with Nature, and Anomie. Transcendence reflects a belief in a higher power, providing comfort and resilience. Harmony with Nature assesses the individual’s perceived unity with their environment. Anomie measures existential voids or life’s perceived lack of meaning. The scale yields a minimum of 29 and a maximum of 145 points, and as scores increase, spiritual well-being increases. The scale is rated on a 5-point Likert scale. The feeling of belonging to a strong divine entity comforts me. I feel God’s help when I face a problem. My faith shows me a better way of life. My faith and values strengthen my ability to withstand hardships. I think events beyond my control cause my distress. In the study conducted by Ekşi and Kardaş, the Cronbach’s Alpha value was found to be 0.88 (Ekşi & Kardaş, 2017). In this study, the Cronbach’s Alpha value was determined to be 0.90.

The Spiritual Well-Being Scale used in the study consists of three subscales: Transcendence, Harmony with Nature, and Anomie. The analyses were conducted at the subscale level, considering the contamination risk highlighted by Koenig and Carey (2024). Particular attention was given to the Anomie subscale, as it may overlap with psychological constructs.

### Beck Hopelessness Scale

The Beck Hopelessness Scale was developed by Beck et al. (Beck et al., 1974). The validity and reliability studies of the scale in our country were conducted by Durak and Palabıyıkoğlu in (1994). (Durak & Palabiyikoğlu, 1994) The Beck Hopelessness Scale consists of 20 true–false items grouped into three main sub-dimensions. Feelings about the Future: This sub-dimension captures individuals’ negative expectations and pessimistic beliefs about their future, reflecting a sense of despair. Loss of Motivation: This aspect assesses the reduction in motivation and willingness to make efforts toward future goals, often due to a belief that these efforts will not yield positive outcomes. Lack of Future Expectations: This sub-dimension evaluates a general absence of hope and expectation for positive developments in future. Eleven of these statements are true, and nine are false. Each correct answer receives 1 point, each incorrect answer receives 0 points, and the total score ranges from 0 to 20. The sum of the scores forms the hopelessness score of 28. A high score on the scale indicates a high level of hopelessness. The Cronbach’s Alpha value in the study conducted by Beck et al. was found to be 0.93. (Beck et al., 1974) In this study, the Cronbach’s Alpha value was determined to be 0.84.

### Data Collection

The data were collected from eligible patients in the Cardiology Unit and ICU between December 2022 and February 2023. The data were collected by collaborating with the Coronary ICU and Cardiology Service team. Face-to-face interviews with patients were obtained at times that did not coincide with their meals, sleep, or visiting hours. The data collection process took approximately 5–10 min. The data were collected after the patients had stabilized following coronary angiography and were able to provide appropriate answers to the questions. After explaining the purpose of the research and answering the questions, verbal and written consent was obtained from patients and their families in the ward and patients alone in the ICU before data collection forms were handed to the patients to be completed by marking their preferred choices individually.

#### Data Evaluation

Data analysis was conducted using the SPSS 22 package program. Skewness, Kurtosis values, and histogram graphs were used to determine whether the measurements showed normal distribution. It was determined that the Transcendence subscale did not show normal distribution. It was found that the other measurements showed normal distribution. Analyses were carried out accordingly. Cronbach’s Alpha value was used to test the reliability of the scales. Frequency and percentage analyses were used to determine the descriptive characteristics. Independent samples *t* test and Mann–Whitney U test were used to compare the means of two scores. One-way ANOVA and Kruskal–Wallis tests were used to compare the means of more than two scores. Correlation analysis was used to determine the relationship between measurements. The significance level was accepted as 0.05. The data analysis was conducted with the support of a nurse academic who had previously received training in statistical analysis.

#### Ethical Approval of the Study

Ethical approval was obtained from the Atatürk University Faculty of Medicine Ethics Committee to conduct the study. Institutional permission was obtained from the Atatürk Provincial Health Directorate, where the research was conducted. In addition, verbal consent was obtained from the participants who met the research criteria and agreed to participate by explaining the purpose of the study and answering their questions. Patients were informed that the data collected during the research would be processed confidentially and would not be used for any other purpose and that they could withdraw from the study at any time if they wished.

## Results

The distribution of individuals who have experienced myocardial infarction among the sub-sections of the preservation units to examine the relationship between spiritual well-being and those caused by hopelessness. It was determined that 70.9% of the patients were male, 51% were in the 20–59 age range, 48.3% had a BMI in the 25–29.9 range, 97.4% were married, 64.2% had a nuclear family, and 64.2% practiced their religious duties always when healthy (Table 1).

**Table 1:** Distribution According to Patients’ Sociodemographic Characteristics

Characteristics	Variables	N	%
Gender	Female	44	29.1
	Male	107	70.9
Age	20–59	77	51
	60 and above	74	49
BMI	18.5–24.9 (normal)	41	27.2
	25–29.9 (overweight)	73	48.3
	30 and above (Obese)	37	24.5
Marital status	Married	147	97.4
	Single	4	2.6
Family type	Nuclear	97	64.2
	Extended	54	35.8
Religious practices while healthy	Always	97	64.2
	Often	17	11.3
	Sometimes	29	19.2
	Never	8	5.3

Upon examination of the mean scores, it was determined that the average score for Transcendence was 70.60 ± 5.53, for Harmony with Nature was 30.52 ± 4.32, and for Anomie was 16.87 ± 5.51. Additionally, the average score for effect was 0.70 ± 1.14, for Loss of Motivation was 2.89 ± 1.35, for Expectations was 1.73 ± 1.29, and for Beck Hopelessness Scale was 5.70 ± 3.41 (Table 2).

**Table 2:** Mean Scores on the Spiritual Well-Being Scale and Beck Hopelessness Scale and Subscales for Patients

Scales	x̄ ± SD	Min	Max
Transcendence	70.60 ± 5.53	42	75
Harmony with Nature	30.52 ± 4.32	12	35
Anomie	16.87 ± 5.51	7	32
Spiritual Well-Being Scale	126.25 ± 12.35	83	144
Affect	0.70 ± 1.14	0	5
Loss of Motivation	2.89 ± 1.35	0	7
Expectations	1.73 ± 1.29	0	5
Beck Hopelessness Scale	5.70 ± 3.41	2	18

It was determined that the scores of the Harmony with Nature dimension were higher among those with a body mass index of 25–29.9 compared to those with a BMI of 30 and above, which was significantly associated *(p* < *0.05).* It was determined that the scores of the Transcendence dimension were higher among married individuals than single individuals, and this difference was statistically significant *(p* < *0.05).* It was found that those who always fulfilled their religious obligations while healthy were significantly associated with higher scores in the Transcendence and Harmony with Nature dimensions than those who fulfilled them frequently. This difference was statistically significant *(p* < *0.05)* (Table 3). It was determined that those who always fulfilled their religious obligations. At the same time, healthy had lower scores in the Anomie dimension than those who fulfilled them sometimes, which was statistically significant *(p* < *0.05).* It was found that those who always fulfilled their religious obligations while healthy had higher scores on the Spiritual Well-Being Scale compared to others, and this difference was statistically significant *(p* < *0.05)* (Table 3).

**Table 3:** Comparison of Spiritual Well-Being Scale and Subscale Mean Scores by Patients’ Sociodemographic Characteristics

Characteristics	Transcendence	Harmony with Nature	Anomie	Spiritual Well-Being Scale
	x̄ ± SD	x̄ ± SD	x̄ ± SD	x̄ ± SD
*Gender* Female Male	70.82 ± 4.92	30.23 ± 4.79	17.89 ± 5.62	125.16 ± 12.10
	70.51 ± 5.78	30.64 ± 4.13	16.45 ± 5.43	126.70 ± 12.48
Test, p value	*U* = 2330.50 *p* = 0.923	*t* = − 0.526 *p* = 0.600	*t* = 1.463 *p* = 0.145	*t* = − 0.696 *p* = 0.488
*Age* 20–59 60 and above	70.25 ± 5.59	30.97 ± 4.10	16.94 ± 5.50	126.29 ± 12.02
	70.97 ± 5.47	30.04 ± 4.52	16.80 ± 5.55	126.22 ± 12.77
*Test, p value*	*U* = 2578.00 *p* = 0.310	*t* = 1.330 *p* = 0.185	*t* = 0.153 *p* = 0.878	*t* = 0.034 *p* = 0.973
*BMI* 18.5–24.9 (normal)^1^ 25–29.9 (overweight)^2^ 30 and above (Obese)^3^	70.54 ± 5.40	30.54 ± 3.79	16.83 ± 5.07	126.24 ± 11.72
	71.31 ± 4.70	31.26 ± 4.09	16.52 ± 6.03	128.05 ± 11.88
	69.27 ± 6.92	29.03 ± 5.00	17.59 ± 4.92	122.70 ± 13.47
*Test, p value, and difference*	KW = 3.919 *p* = 0.141	***F* = 3.383** ***p* = 0.037** **2–3**	*F* = 0.465 *p* = 0.629	*F* = 2.346 *p* = 0.099
*Marital status* Married Single	70.80 ± 5.20	30.57 ± 4.34	16.72 ± 5.39	126.65 ± 12.00
	63.25 ± 11.62	28.50 ± 3.70	22.25 ± 7.97	111.50 ± 17.90
*Test, p value*	***U* = 102.00** ***p* = 0.025**	*t* = − 0.543 *p* = 0.588	*t* = − 1.214 *p* = 0.227	*t* = − 0.767 *p* = 0.444
*Family type* Nuclear Extended	70.09 ± 6.39	30.39 ± 4.31	16.95 ± 5.85	125.54 ± 13.44
	71.52 ± 3.34	30.74 ± 4.37	16.72 ± 4.87	127.54 ± 10.09
*Test, p value*	*U* = 2478.00 *p* = 0.581	*t* = − 0.474 *p* = 0.636	*t* = 0.241 *p* = 0.810	*t* = − 0.954 *p* = 0.342
*Religious practices while healthy* Always^1^ Often^2^ Sometimes^3^ Never^4^	72.15 ± 3.62	31.27 ± 4.16	15.68 ± 4.66	129.74 ± 9.69
	67.65 ± 6.05	28.53 ± 4.72	18.94 ± 5.68	119.24 ± 15.13
	69.62 ± 5.24	29.79 ± 4.03	18.76 ± 6.15	122.66 ± 10.79
	61.63 ± 11.41	28.25 ± 4.68	20.00 ± 8.47	111.88 ± 20.35
*Test, p value, and difference*	KW = 30.539 *p* = 0.001 1–2	*F* = 3.328 *p* = 0.021 1–2	*F* = 4.620 *p* = 0.001 1–3	*F* = 10.524 *p* = 0.001 1–2, 1–3, 1–4

It was determined that the average scores of Emotions, Expectations, and Beck Hopelessness Scale sub-dimensions were low for those with a body mass index of 25–29.9 compared to those with 30 and above, and this difference was statistically significant *(p* < *0.05).* It was determined that the average scores of the Motivation Loss sub-dimension were higher for those with extended family than those with nuclear family. This difference was statistically significant *(p* < *0.05)* (Table 4).

**Table 4:** Comparison of Beck Hopelessness Scale and Sub-dimension Scores According to Patients’ Sociodemographic Characteristics

Characteristics	Variables	Affect	Loss of Motivation	Expectations	Beck Hopelessness Scale
		x̄ ± SD	x̄ ± SD	x̄ ± SD	x̄ ± SD
*Gender* Female Male		0.66 ± 1.12	2.73 ± 1.23	1.89 ± 1.33	5.82 ± 3.23
		0.72 ± 1.16	2.95 ± 1.40	1.66 ± 1.27	5.66 ± 3.49
*Test, p value*		*t* = − 0.295 *p* = 0.768	*t* = − 0.935 *p* = 0.351	*t* = 0.964 *p* = 0.337	*t* = 0.252 *p* = 0.801
*Age* 20–59 60 and above		0.66 ± 1.17	2.91 ± 1.32	1.66 ± 1.28	5.58 ± 3.45
		0.74 ± 1.12	2.86 ± 1.39	1.80 ± 1.30	5.84 ± 3.39
*Test, p value*		*t* = − 0.434 *p* = 0.665	*t* = 0.201 *p* = 0.841	*t* = − 0.641 *p* = 0.522	*t* = − 0.445 *p* = 0.649
*BMI* 18.5–24.9 (normal)^1^ 25–29.9 (overweight)^2^ 30 and above (Obese)^3^		0.61 ± 1.05	2.90 ± 1.18	1.68 ± 1.49	5.56 ± 2.67
		0.51 ± 1.01	2.73 ± 1.35	1.49 ± 1.33	4.99 ± 3.32
		1.19 ± 1.35	3.19 ± 1.51	2.24 ± 1.30	7.30 ± 3.84
*Test, p value, and difference*		***F* = 4.799** ***p* = 0.010** **2–3**	*F* = 1.459 *p* = 0.236	***F* = 4.370** ***p* = 0.014** **2–3**	***F* = 6.081** ***p* = 0.003** **2–3**
*Marital status* Married Single		0.69 ± 1.14	2.88 ± 1.35	1.71 ± 1.29	5.67 ± 3.40
		1.00 ± 1.41	3.25 ± 1.26	2.50 ± 1.29	7.00 ± 4.08
*Test, p value*		*t* = − 0.528 *p* = 0.598	*t* = − 0.543 *p* = 0.588	*t* = − 1.214 *p* = 0.227	*t* = − 0.767 *p* = 0.444
*Family type* Nuclear Extended		0.78 ± 1.26	3.09 ± 1.44	1.78 ± 1.39	6.05 ± 3.76
		0.56 ± 0.88	2.52 ± 1.09	1.63 ± 1.10	5.09 ± 2.59
*Test, p value*		*t* = 1.299 *p* = 0.196	***t* = 2.55** ***p* = 0.012**	*t* = 0.701 *p* = 0.484	*t* = 1.845 *p* = 0.067
*Religious practices while healthy* Always^1^ Often^2^ Sometimes^3^ Never^4^		0.49 ± 0.91	2.63 ± 1.08	1.60 ± 1.20	5.052.74
		1.00 ± 1.54	3.53 ± 1.91	1.47 ± 1.37	6.59 ± 4.50
		1.00 ± 1.25	3.07 ± 1.33	2.07 ± 1.39	6.55 ± 3.51
		1.50 ± 1.69	4.00 ± 2.00	2.63 ± 1.51	8.75 ± 5.37
*Test, p value, and difference*		***F* = 3.587** ***p* = 0.015** **1–3, 1–4**	***F* = 4.797** ***p* = 0.003** **1–4**	*F* = 2.597 *p* = 0.055	***F* = 4.602** ***p* = 0.004** **1–4**

It was determined that the average scores of the Emotions sub-dimension were low for those who consistently perform their religious obligations when healthy compared to those who sometimes or never perform them. This difference was statistically significant *(p* < *0.05).* It was also determined that the average scores of the Motivation Loss and Beck Hopelessness Scale sub-dimensions were low for those who consistently perform their religious obligations when they are healthy compared to those who never perform them. This difference was statistically significant *(p* < *0.05)* (Table 4).

The correlation strength was evaluated as weak for 0.10–0.29, moderate for 0.30–0.49, and strong for 0.50–1. A strong negative correlation was observed between Spiritual Well-Being and the Beck Hopelessness Scale (*p* < 0.05). The transcendent subscale demonstrated a significant negative association with hopelessness levels. The transcendent subscale is a unique component as it is unaffected by mental health indicators, making it particularly important in understanding the relationship between spiritual well-being and hopelessness levels. These findings suggest that transcendent aspects of spiritual well-being should be prioritized over total scores when evaluating its impact on hopelessness (Table 5). The analysis revealed that the Transcendence and Harmony with Nature subscales of the Spiritual Well-Being Scale showed a significant negative relationship with hopelessness. In contrast, the Anomie subscale exhibited a significant positive relationship (*p* < 0.05). This finding indicates a potential overlap between the Anomie subscale and psychological constructs, which should be interpreted cautiously. However, evaluating all subscales together provides a comprehensive understanding of the relationship between spiritual well-being and hopelessness.

**Table 5:** Correlation Analysis Results for Patients’ Spiritual Well-Being Scale, Beck Hopelessness Scale, and Sub-Dimensions

	Transcendence		Harmony with Nature	Anomie	Spiritual Well-Being Scale	Affect	Loss of Motivation	Expectations	Beck Hopelessness Scale
Transcendence	*r*	1	0.588^**^	− 0.457^**^	0.856^**^	− 0.514^**^	− 0.399^**^	− 0.383^**^	− 0.544^**^
	*p*		**0.000**	**0.000**	**0.000**	**0.000**	**0.000**	**0.000**	**0.000**
Harmony with Nature	*r*	0.588^**^	1	− 0.362^**^	0.774^**^	− 0.321^**^	− 0.245^**^	− 0.326^**^	− 0.381^**^
	*p*	**0.000**		**0.000**	**0.000**	**0.000**	**0.002**	**0.000**	**0.000**
Anomie	*r*	− 0.457^**^	− 0.362^**^	1	− 0.777^**^	0.468^**^	0.391^**^	0.546^**^	0.578^**^
	*p*	**0.000**	**0.000**		**0.000**	**0.000**	**0.000**	**0.000**	**0.000**
Spiritual Well-Being Scale	*r*	0.856^**^	0.774^**^	− 0.777^**^	1	− 0.551^**^	− 0.439^**^	− 0.529^**^	− 0.634^**^
	*p*	**0.000**	**0.000**	**0.000**		**0.000**	**0.000**	**0.000**	**0.000**
Affect	*r*	− 0.514^**^	− 0.321^**^	0.468^**^	− 0.551^**^	1	0.623^**^	0.488^**^	0.872^**^
	*p*	**0.000**	**0.000**	**0.000**	**0.000**		**0.000**	**0.000**	**0.000**
Loss of Motivation	*r*	− 0.399^**^	− 0.245^**^	0.391^**^	− 0.439^**^	0.623^**^	1	0.323^**^	0.790^**^
	*p*	**0.000**	**0.002**	**0.000**	**0.000**	**0.000**		**0.000**	**0.000**
Expectations	*r*	− 0.383^**^	− 0.326^**^	0.546^**^	− 0.529^**^	0.488^**^	0.323^**^	1	0.736^**^
	*p*	**0.000**	**0.000**	**0.000**	**0.000**	**0.000**	**0.000**		**0.000**
Beck Hopelessness Scale	*r*	− 0.544^**^	− 0.381^**^	0.578^**^	− 0.634^**^	0.872^**^	0.790^**^	0.736^**^	1
	*p*	**0.000**	**0.000**	**0.000**	**0.000**	**0.000**	**0.000**	**0.000**	

^**^. Correlation is Significant at the 0.01 Level (2-Tailed)

## Discussion

The findings of this study indicate a strong and negative relationship between spiritual well-being and hopelessness levels in individuals who have had myocardial infarction. To alleviate the concern of a tautological relationship, it is essential to emphasize that the concepts of spiritual well-being and hopelessness reflect different aspects of mental health. Spiritual well-being is associated with a broader existential framework, including individuals’ beliefs about the meaning of life, connectedness to a higher power, and Harmony with Nature. Hopelessness, in contrast, is associated with a more specific and future-oriented construct, focusing on negative expectations about the future, loss of motivation, and feelings of hopelessness. In contrast, hopelessness is a more specific and future-oriented construct, focusing on negative expectations about the future, loss of motivation, and feelings of hopelessness. Therefore, the observed relationship between these two variables is not a repetition; it reflects the interaction of different dimensions of mental well-being. It is thought that high levels of spiritual well-being may be associated with lower levels of pessimism and hopelessness. When the literature was reviewed, it was observed that there were limited studies on the hopelessness levels of individuals who had experienced a myocardial infarction (Savaşan et al., 2013; Yaman & Muz, 2021), while there were studies on spiritual well-being with different sample groups but no specific studies on individuals who had experienced a myocardial infarction. In this context, it is thought that the data obtained from the study will contribute to filling this gap.

In the study, it was found that the transcendent subscale of spiritual well-being, which reflects the belief that everything comes from Allah, was particularly high in individuals who had suffered a myocardial infarction (Table 2). Unlike the total spiritual well-being scores highlighted in other studies, this study emphasizes the transcendent subscale as it is unaffected by mental health indicators. For example, Çiçekli and Çalışkan (2022) found that the overall spiritual well-being levels of patients were above average (Çiçekli & Çalişkan, 2022). Similarly, Bezerre et al. (2018) reported lower mean scores in their investigation of spiritual well-being in patients before cardiac surgery (Bezerra et al., 2018), while Aşiret and Okatan (2019) observed higher total spiritual well-being levels in hypertensive patients compared to this study (Aşiret & Okatan, 2019). These differences highlight how focusing on total scores can yield varying results across patient populations and cultural contexts. By contrast, this study’s emphasis on the transcendent subscale provides a unique perspective, offering insights independent of mental health-related constructs.

In this study, it was found that the Beck hopelessness levels of patients who had experienced MI were low (Table 2). Similarly, in a study conducted by Yaman and Muz to determine the hopelessness level in individuals who had experienced myocardial infarction, it was found that the hopelessness level was low (Yaman & Muz, 2021); however, Savaşan et al. ( 2013) examined the hopelessness level in elderly patients, which focused on the hopelessness levels of coronary artery patients, which was found to be higher. In the study of Akçay Fırat and Dedeli, it was determined that patients who had MI experienced moderate hopelessness (Arslantaş et al., 2010). Karakurt et al.’s study determined that the hopelessness level of heart patients was above the moderate level (Karakurt et al., 2018). It is encouraging that the hopelessness level of the patients in this study is low. The short duration of diagnosis, the rapid relief of fear of death and severe symptoms, and the quick return to everyday life may have contributed to the low level of hopelessness. Therefore, nurses should follow the process of the patient groups they provide care for and emphasize the importance of implementing necessary nursing interventions to reduce hopelessness.

There are significant differences between the level of spiritual well-being of the patients who participated in the study and their BMI, marital status, and their level of fulfilling religious obligations when healthy. There is a statistically significant relationship between the BMI of overweight patients (25–29.9) and their level of spiritual well-being compared to those who are obese (30 and above). Harmony with Nature is higher in overweight patients (25–29.9) than in those who are obese at degree 1 (30 and above) (Table 3). Harmony with Nature, an inseparable part of spirituality, is the individual’s knowledge of and adaptation to their physical environment. It is also the individual’s contemplation of their relationship with themselves, nature, and the Creator. (Ekşi & Kardaş, 2017). It can be said that overweight individuals are more lively and harmonious with nature and themselves compared to those who are obese.

The transcendence sub-dimension of married individuals is higher than that of singles. Transcendence is believing in a solid and infinite existence and being weak and needy toward it. In a similar study to this research, Uğurluoğlu and Erdem found that the level of transcendence of married individuals who have experienced trauma was higher than that of singles (Uğurluoğlu & Erdem, 2019). In Nurkan’s study, the level of spiritual well-being of married individuals was found to be higher and statistically significant than that of single (Nurkan, 2020). In Çiçekli’s study conducted with patients who were scheduled to undergo open-heart surgery, the average score of married individuals was found to be higher than that of singles (Çiçekli & Çalişkan, 2022). These results suggest that the spiritual well-being of married individuals is more substantial, and they believe more in the existence of divine power and the transience of worldly life than singles. This situation may be attributed to married individuals having more responsibilities than singles.

The study found that individuals who perform their religious duties regularly when healthy have significantly higher levels of transcendence, Harmony with Nature, and spiritual well-being than others. In comparison, the anomic subgroup had lower levels. Fulfilling religious duties may provide inner peace to individuals. This situation may be related to the cultural and religious structures of the individuals. Significant differences were observed in the Beck Despair Scale levels of the participating patients according to their BMI, family type, and frequency of performing religious duties when healthy.

Regarding the despair scale and its subscales, individuals with a BMI of 25–29.9 were significantly associated with lower levels of despair related to their expectations and feelings about the future than those with a BMI of 30 and above. Research indicates that an increase in weight leads to a decrease in physical activity level, exacerbation of symptoms during illness, and triggering of complications, which increase the level of despair (Roberts et al., 2000; Salık & Sarıtaş, 2021). However, some studies do not support the research findings. In Salik’s study conducted on patients with heart failure and despair levels, the average despair scores of those who were overweight were found to be higher than those who were obese (Salık & Sarıtaş, 2021). In Anatolian culture and many other cultures, being overweight is seen as a sign of wealth, health, happiness, and peace (Satman, 2012). The difference between studies is thought to be due to differences in the weight perception resulting from sociocultural differences among the sample groups.

It was determined that the average hopelessness scores of singles were higher than those of married individuals but statistically insignificant (Table 4). Similarly, it was found that the hopelessness levels of singles were higher than those of married individuals but statistically insignificant (Tercanli & Demir, 2012). However, different results from this study can also be seen in the literature. In a study conducted on the hopelessness level of patients with heart failure, it was found that the hopelessness level of married individuals was higher than that of singles and statistically significant (Salık & Sarıtaş, 2021). The fact that marriage has an impact on many aspects of an individual’s life, the feeling of loneliness is reduced, and there is love, respect, togetherness, and cooperation, as well as making joint decisions and motivations for the future, suggests that it reduces hopelessness.

It has been observed that individuals from nuclear families have higher levels of motivation loss and hopelessness compared to those from extended families. Similarly, in a study by Demirel et al. on the hopelessness levels of cancer patients according to metastasis, the average scores of individuals from nuclear families were found to be higher than those from extended families (Demirel, et al., 2015). However, in a different study on the Beck Hopelessness Scale and various variables in Gümüşhane province, the average hopelessness score of individuals from nuclear families was lower than that of those from extended families but statistically insignificant (Tercanli & Demir, 2012). It is believed that the majority of the age group studied in this research had grandchildren and did not experience loneliness as they lived with their daughters-in-law, grandchildren, sons-in-law, and children, which resulted in lower levels of hopelessness due to their love for their grandchildren. When the studies are examined, it is thought that the differences between them may be due to differences in age range, cultural differences, and ethnic and religious background in the samples studied.

It was observed that individuals who regularly performed their religious obligations. At the same time, healthy people were more emotionally fulfilled and had lower motivation loss and hopelessness levels than those who never performed their religious duties (Table 4). A study on the relationship between regular prayer and mental health showed that individuals who perform regular prayers are better able to cope with situations such as hopelessness, loneliness, and stress and are mentally healthier (Gök, 2019). It was shown that religious beliefs and practices change a person’s perspective in adverse situations, increase their optimism and hope levels, and make them feel bad moments lighter with positive thoughts for the future (Gashi, 2020).

When the research findings are examined, a strong and negative correlation between spiritual well-being and hopelessness levels is observed (Table 5). The increase in spiritual well-being in patients indicates a decrease in their levels of hopelessness. This situation can be attributed to the high spirituality of patients who believe they have found the meaning and purpose of their lives and have high levels of hope and motivation free from negative thoughts. This finding is encouraging. In their study, Gholamhosseini et al. showed that spiritual counseling can significantly affect the level of hope in patients with myocardial infarction, and patients who received spiritual counseling in the follow-up were substantially more hopeful (Gök, 2019). It is believed that higher levels of spiritual well-being are associated with reduced hopelessness and improved parameters such as acceptance of illness, quality of life, motivation, and joy of life. Nurses have essential duties in this field.

The findings demonstrate a strong negative relationship between spiritual well-being and hopelessness. However, considering the concerns raised by Koenig and Carey (2024) and Bambling (2024) regarding the potential contamination of specific psychometric scales, the positive relationship between the Anomie subscale and hopelessness may suggest an overlap with psychological health constructs (Koenig & Carey, 2024). Despite this, including all subscales in the analysis supports the validity of the overall relationship. Future studies should utilize measurement tools that distinctly separate spiritual well-being and psychological constructs for more robust findings.

### Limitations of the Study

The study’s limitations include the fact that the data were collected from a single hospital was limited to a specific date and 151 patients, and no comparison was made with other institutions. The results obtained from the study can only be generalized to the hospital where the study was conducted. This cross-sectional study prevents any causal inference regarding the associations identified.

The Anomie subscale of the Spiritual Well-Being Scale may overlap with psychological constructs, as suggested by its positive association with hopelessness. While this overlap could influence the interpretation of results, the study included all subscales to ensure a holistic evaluation of spiritual well-being. Future studies should address this limitation by employing scales that distinctly separate spirituality from psychological well-being.

## Conclusion and Recommendations

This study aimed to examine the relationship between spiritual well-being and hopelessness levels in individuals who had suffered a myocardial infarction. The following conclusions were made: Participants demonstrated high levels of spiritual well-being and low levels of hopelessness. A statistically significant relationship was found between the Spiritual Well-Being Scale and the Beck Hopelessness Scale in relation to BMI, marital status, family type, and regular performance of religious duties. No statistically significant relationship was observed between the Spiritual Well-Being Scale or the Beck Hopelessness Scale and gender or age. A strong negative relationship was identified between spiritual well-being and hopelessness levels.

This study emphasizes the transcendent subscale as a meaningful aspect of spiritual well-being, showing its strong association with lower hopelessness levels. Integrating spiritual counseling into nursing interventions, focusing particularly on transcendent elements, is recommended to enhance psychological resilience. Interpretations should prioritize transcendent subscale results over total scores of spiritual well-being, considering potential overlaps with mental health indicators.

Screening for spiritual well-being levels and subsequent determination of hopelessness levels in individuals who have suffered a myocardial infarction is essential. Integration of spiritual care into individualized nursing interventions is recommended to address the spiritual well-being levels of patients with high levels of hopelessness, aiming to improve their overall psychological resilience and coping mechanisms. Spiritual counseling should be provided at various stages of treatment—before, during, or after—given its association with patient participation in areas such as treatment adherence, nutrition, health efforts, and sleep patterns. Nursing interventions are planned to ensure that patients perform their religious duties to the extent possible and within their means, as this may increase their hope levels.

This study identified a strong negative relationship between spiritual well-being and hopelessness. However, it is essential to acknowledge that some subscales, particularly Anomie, may overlap with psychological constructs, as noted in the literature. Researchers should consider alternative scales that clarify spiritual and psychological constructs in future studies to ensure more precise findings.

### Clinical to Relevance

The findings suggest that higher levels of spiritual well-being are associated with lower levels of hopelessness in patients after myocardial infarction. This situation reveals the importance of spiritual support in nursing practice. For example, spiritual counseling may encourage patients to strengthen their sense of purpose and resilience, which is associated with reduced feelings of hopelessness. It is important to note that this suggestion is based on the relationship observed in the study and does not imply a direct causal effect. Nevertheless, it is thought that spiritual interventions, such as spiritual counseling or access to religious practices, can be used as a complementary method in holistic nursing care. By addressing patients’ spiritual and psychological needs, nurses can improve myocardial infarction patients’ emotional resilience, motivation, and overall quality of life.
